# Cisplatin treatment induces attention deficits and impairs synaptic integrity in the prefrontal cortex in mice

**DOI:** 10.1038/s41598-018-35919-x

**Published:** 2018-11-27

**Authors:** XiaoJiao Huo, Teresa M. Reyes, Cobi J. Heijnen, Annemieke Kavelaars

**Affiliations:** 1 Neuroimmunology Laboratory, Department of Symptom Research, The University of Texas MD Anderson Cancer Center, Houston, TX USA; 2 Department of Psychiatry and Behavioral Neuroscience, College of Medicine, University of Cincinnati, Cincinnati, OH USA

## Abstract

Patients treated for cancer frequently experience chemobrain, characterized by impaired memory and reduced attention. These deficits often persist after treatment, and no preventive or curative interventions exist. In mice, we assessed the effect of cisplatin chemotherapy on attention using the 5-choice serial reaction time task and on synaptic integrity. We also assessed the capacity of mesenchymal stem cells to normalize the characteristics of chemobrain. Mice were trained in the 5-choice serial reaction time task. After reaching advancement criteria at a 4-second stimulus time, they were treated with cisplatin followed by nasal administration of mesenchymal stem cells. Cisplatin reduced the percentage of correct responses due to an increase in omissions, indicating attention deficits. Mesenchymal stem cell treatment reversed these cisplatin-induced deficits in attention. Cisplatin also induced abnormalities in markers of synaptic integrity in the prefrontal cortex. Specifically, cisplatin decreased expression of the global presynaptic marker synaptophysin and the glutamatergic presynaptic marker vGlut2. Expression of the presynaptic GABAergic marker vGAT increased. Nasal mesenchymal stem cell administration normalized these markers of synaptic integrity. In conclusion, cisplatin induces long-lasting attention deficits that are associated with decreased synaptic integrity in the prefrontal cortex. Nasal administration of mesenchymal stem cells reversed these behavioural and structural deficits.

## Introduction

The American Cancer Society estimates that the number of cancer survivors in the United States will increase from more than 15.5 million in 2016 to more than 20 million by 2026^1^. Inevitably, the number of cancer survivors dealing with the long-term adverse effects of cancer and its treatment will increase. For example, cognitive deficits, including difficulties with attention, concentration, processing speed, and language, are experienced by up to 75% percent of patients treated with chemotherapy for cancers outside the nervous system^2–7^. These cognitive deficits, also known as “chemobrain,” can persist for months or even years after completion of cancer treatment. Advanced neuroimaging techniques show global disruptions of connectivity in patients suffering from chemotherapy-induced cognitive impairment, along with structural alterations in white and grey matter^8–12^. To date, no interventions to prevent or reverse these adverse effects of cancer and its treatment have been approved by the US Food and Drug Administration.

Most studies on chemobrain in humans have focused on women treated for breast cancer. However, emerging evidence indicates that cognitive deficits also affect patients treated with platinum-based compounds for small cell lung carcinoma, bladder cancer, prostate cancer, or ovarian cancer^13–18^. Advanced imaging techniques confirm functional and structural abnormalities in the brains of patients treated with platinum-based compounds^14,16–18^.

Platinum-based compounds induce cognitive deficits and damage to the brain in rodents as well^19–22^. We recently showed that cisplatin treatment of young adult mice (10‒12 weeks old) impairs their performance in behavioural tasks examining short-term memory and spatial orientation, such as the novel object and place recognition task (NOPRT), and spontaneous alternations in the Y-maze^21,22^. These cognitive deficits were not associated with reductions in locomotor activity, food intake, total interaction times in the NOPRT, or total number of arm entries in the Y-maze, indicating that the deficit cannot be explained by a general decrease in activity^21,22^. More importantly, we showed that intranasal application of mesenchymal stem cells (MSCs) 48 and 96 hours after completion of cisplatin treatment completely reversed these cisplatin-induced memory and orientation deficits in young adult mice (Chiu *et al*.^23^).

As mentioned above, patients with chemobrain not only show signs of memory deficits, but also show impairment in attention and executive functioning. The 5-choice serial reaction time task (5CSRTT) has been developed to assess attention deficits in rats and mice^24–28^. We used this task in adult mice (7‒8 months old) to examine the effects of cisplatin treatment on these translationally relevant aspects of chemotherapy-induced deficits in cognitive function. We also assessed the effects of nasal MSC application on performance in the 5CSRTT. To get more insight into the structural abnormalities associated with impaired performance in this behavioural task, we examined the effects of cisplatin and MSCs on synaptic integrity in the prefrontal cortex.

## Methods

### Animals

The animal experiments reported herein were conducted in accordance with ARRIVE (Animal Research: Reporting of *In Vivo* Experiments) guidelines for ethical animal research and with National Institutes of Health guidance on the care and use of laboratory animals. All experiments were conducted at The University of Texas MD Anderson Cancer Center in Houston, Texas. All procedures were approved by the MD Anderson Institutional Animal Care and Use Committee.

Experiments were performed with male C57BL/6J mice (Jackson Laboratory, Bar Harbor, ME) housed at 22 ± 2 °C, on a 12/12-hour reverse dark–light cycle (dark 830–2030 hours) with water and food *ad libitum*. Mice were group-housed for the duration of the study. At an age of 5‒6 months, all mice were food restricted to 80–85% of their free-feeding body weight, as is standard for operant testing protocols. Mice were trained in the operant chamber as described below until all mice had reached the 4-second stimulus time advancement criteria. This approach was selected to ensure that all mice had learned the task before exposure to cisplatin. Mice were then returned to free feeding and treated with 2 cycles of 5 daily intraperitoneal injections of cisplatin (2.3 mg/kg/day; Fresenius Kabi USA, Lake Zurich, IL) or phosphate-buffered saline (PBS), followed by a 5-day rest without injections. This schedule was selected on the basis of our previous work^21^.

### Experimental Plan

We performed 2 separate experiments. In Experiment 1 (saline n = 5 mice; cisplatin n = 7 mice), there were no further interventions. In Experiment 2, mice were treated with saline (n = 11) or cisplatin (n = 20) followed by nasal administration of MSCs or PBS (saline/PBS: n = 5; saline/MSCs: n = 6; cisplatin/PBS: n = 10; cisplatin/MSCs: n = 10) 48 hours and 96 hours after completion of cisplatin treatment. We have not detected differences between the saline/PBS and saline/MSCs groups; therefore, we pooled the results from these 2 groups for data analysis and labelled the pooled group “control”.

Mouse MSCs (GIBCO Mouse C57BL/6; Invitrogen, Carlsbad, CA) were cultured in Dulbecco’s Modified Eagle’s medium/F12 medium with GlutaMax-I, supplemented with 10% MSC-qualified foetal bovine serum and 5 µg/mL gentamycin (all from Invitrogen). Before administration of MSCs, 3 µL per nostril of hyaluronidase in PBS (total 100 U per mouse; Sigma-Aldrich, St. Louis, MO) was administered to each nostril to increase the permeability of the nasal mucosa^29,30^. Thirty minutes later mice received MSCs applied as 2 doses of 3 µL to each nostril (total volume of 12 µL with 10^6^ cells per mouse per day^29,30^) or PBS. We selected this schedule on the basis of our previous work in models of ischemic brain damage^30–33^. Three days before returning to the 5CSRTT training, mice were put back on the food-restriction schedule.

### 5-Choice Serial Reaction Time Task

**Figure 1:**
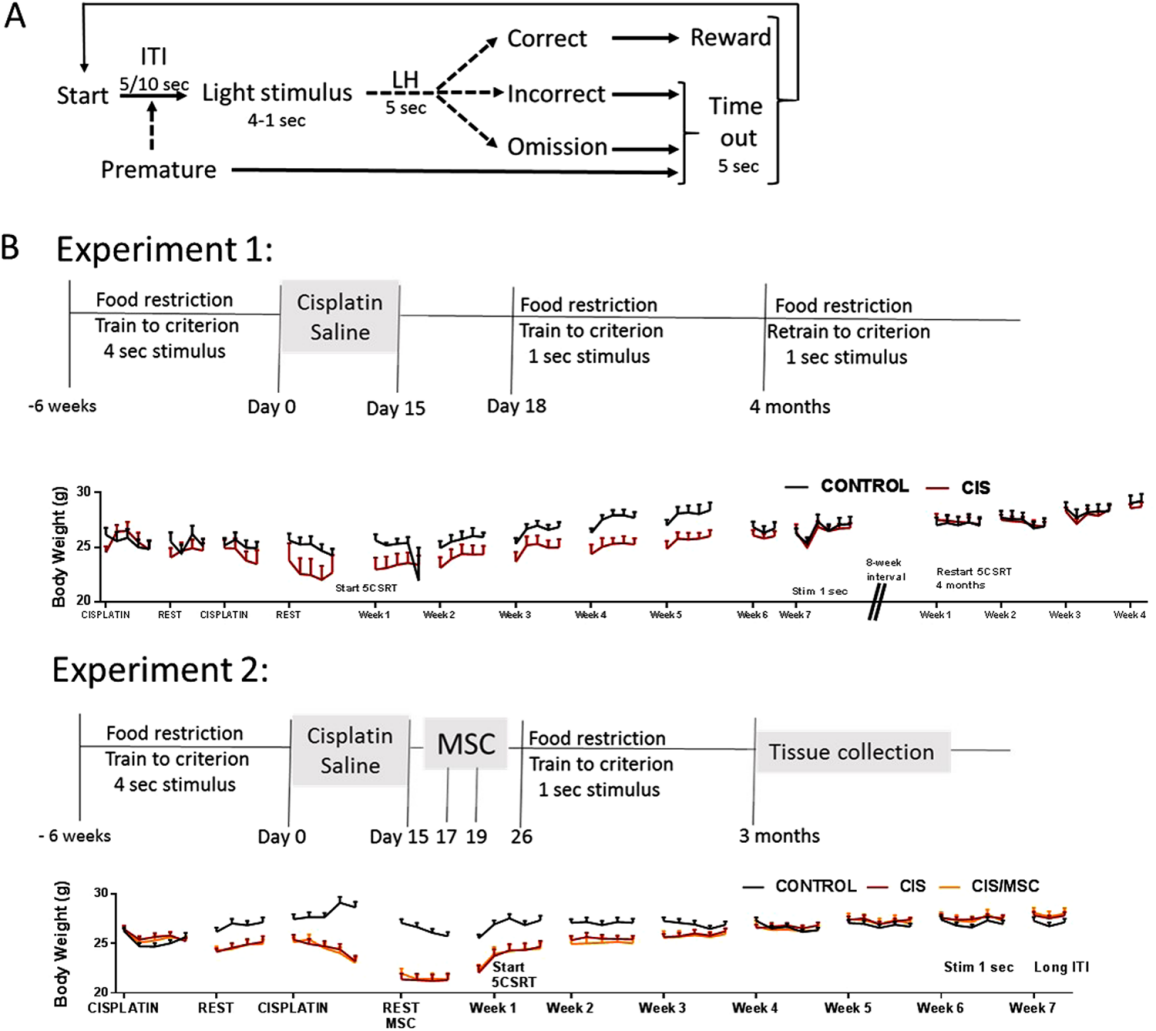
Schematic overview of 5CSRTT and experimental setup. (**A**) Overview of the 5-choice serial reaction time task procedure. (**B**) Schematic overview and body weight over time for the 2 experiments. Cisplatin treatment consisted of 5 days of 2.3 mg/kg i.p, followed by 5 days of rest and another 5 days of cisplatin. Mice were allowed free access to food during the course of treatment. After cisplatin mice were exposed to the 5 CSRT for 6–7 weeks. In experiment 1, training was resumed after a 2 month-interval (4 months after cisplatin). In experiment 2, the long ITI was introduced during the last week of 5CSRT, when performance at the 1 sec stimulus time and 5 second ITI had been stable for at least 5 days. Abbreviations: 5CSRTT, 5-choice serial reaction time task; i.p., intraperitoneal; ITI, intertrial interval; LH, limit hold; MSC, mesenchymal stem cells.

The 5-CSRTT was administered by a single investigator as described previously^28^ and using the schedule presented in Fig. 1A. The operant testing boxes (Modular test chamber, model ENV-307W) were obtained from Med Associates INC (Fairfax, VT). Mice were exposed to 1 session per day from Monday through Friday. They were food restricted from Sunday until end of day on Friday, and then had free access to food until Sunday morning.

#### Chamber Habituation, Nose Poke, and Food Magazine Pre-Training

Before starting 5CSRTT training, mice were exposed to the reward (Chocolate-flavored Dustless Precision Pellets, Med Associates, St Albans, VT) in their home cage. The next day, nose-poke training was started by introducing each mouse individually to the chamber for 20 minutes, with all 5 stimulus lights and the food magazine light illuminated. Two chocolate pellets were placed in each of the 5 light apertures, and 10 chocolate pellets were placed in the magazine. Daily training sessions were repeated until the mouse reliably consumed all pellets provided. During the subsequent food magazine training sessions, a light in 1 of the 5 stimulus holes was kept on until the mouse made a nose poke and consumed the pellet in the food magazine. This session was repeated 2–3 times, until the mouse could finish up to 50 trials (consume 50 pellets) within the 20-minute session time.

#### 5CSRTT Training

During the 5CSRTT training, a nose poke into the illuminated hole was required to obtain the reward. Training was initiated by completion of a magazine entry, triggering an intertrial interval (ITI) of 5–10 seconds during which the animal waited for illumination of 1 of the 5 holes at the back of the chamber (Fig. 1A). A nose poke to the illuminated hole completed a correct trial and triggered delivery of the reward; the cycle was then repeated. The initial stimulus time was 16 seconds. Mice were kept at the same stimulus time for 2 consecutive sessions after meeting the advancement criterion (stimulus time of 16 seconds or 8 seconds: ≥30 correct trials and 50 trials in total over 2 consecutive days; stimulus time ≤4 seconds: >50% correct trials and 100 trials). Then timing was reduced to 8 seconds, 4 seconds, 2 seconds, and 1 second.

Only a correct response during the stimulus time and a following 2-second limit hold time initiated the reward. If the mouse nose poked correctly during the stimulus time plus the 2-second limit hold, then the food reinforcer was delivered. If the mouse nose poked a non-illuminated hole (incorrect response) or failed to respond within the stimulus time plus the 2-second limit hold period (omitted response), the chamber light was illuminated for 5 seconds (time out), after which the cycle restarted. If the mouse nose poked during the 5–10-second ITI (premature response), a 5-second time out was presented. A session was completed when the maximum number of trials (50 trials for the 16-second or 8-second stimulus time, or 100 trials for stimulus times ≤4 seconds), or the maximum session duration (30 minutes) was reached.

After a mouse met advancement criterion at a stimulus time of 4 seconds, we continued training it with a 4-second stimulus time for at least 8 days. Subsequently, the mouse was returned to free feeding, with cisplatin treatment initiated 3 days later. After completion of cisplatin treatment, mice were food restricted for 1 week, after which 5CSRTT was restarted at a stimulus time of 4 seconds. Mice continued the 5CSRTT until they met advancement criteria at a stimulus time of 1 second.

To determine whether there is spontaneous recovery of cisplatin-induced attention deficits, the mice in Experiment 1 were re-tested in the 5CSRTT starting 4 months after completion of treatment, at a 4-second stimulus time. We again tested the mice when they reached advancement criteria at the 1-second stimulus time.

The number and percentage of correct, incorrect, and omitted trials, latency times to correct and incorrect responses, and latency to collect the reward were recorded.

### Immunofluorescence Analysis of Synaptic Markers

Twelve weeks after completion of cisplatin treatment, half of the mice from Experiment 2 were perfused intracardially with ice-cold PBS followed by 4% paraformaldehyde in PBS. Brains were post-fixed in 4% paraformaldehyde for 6 hours, cryoprotected in sucrose, and frozen in optimal cutting temperature compound (O.C.T.; Sakura Finetek, Torrance, CA). Sagittal sections (8 μm) were incubated with the following antibodies: rabbit anti-synaptophysin (1:1000; Millipore), mouse anti-vGLUT2 (1:1000; Abcam, Cambridge, MA), rabbit anti-PSD95 (1:1000; Abcam), mouse anti-vGAT (1:100; Abcam), and rabbit anti-gephyrin (1:1000; Abcam) followed by Alexa-488 goat anti-rabbit (1:1000; Invitrogen, Grand Island, NY) for synaptophysin, Alexa-488 goat anti-mouse (1:500; Invitrogen) for vGLUT2 and vGAT, and Alexa-594 goat anti-rabbit (1:500; Invitrogen) for PSD95 and gephyrin. As a negative control, primary antibody was omitted. Sections were visualized using a Leica SPE confocal microscope. Expression of each synaptic marker was quantified in 3–4 sections per mouse and 4–5 mice per group. The mean intensity of fluorescence was calculated using ImageJ software.

### Statistical analysis

Data are expressed as mean ± standard error of the mean. Statistical analysis was performed using 1-way or 2-way analysis of variance (ANOVA) followed by Tukey analysis. *P* < 0.05 was considered statistically significant.

## Results

### Effect of Cisplatin on Learning/Memory in the 5CSRTT

Before treatment with cisplatin, we trained adult male C57BL/6 mice aged 5‒6 months at the start of the study in the operant chamber until they met advancement criteria at a stimulus time of 4 seconds (see Fig. 1B for schematic overview). Subsequently, mice were treated with 2 cycles of cisplatin (5 days 2.3 mg/kg/day, 5 days rest, 5 days 2.3 mg/kg/day) or vehicle and body weight was monitored regularly (Fig. 1B). In Experiment 1, mice did not receive further treatments. In Experiment 2, half the mice received MSCs via the nasal route 48 and 96 hours after completion of cisplatin treatment.

**Figure 2:**
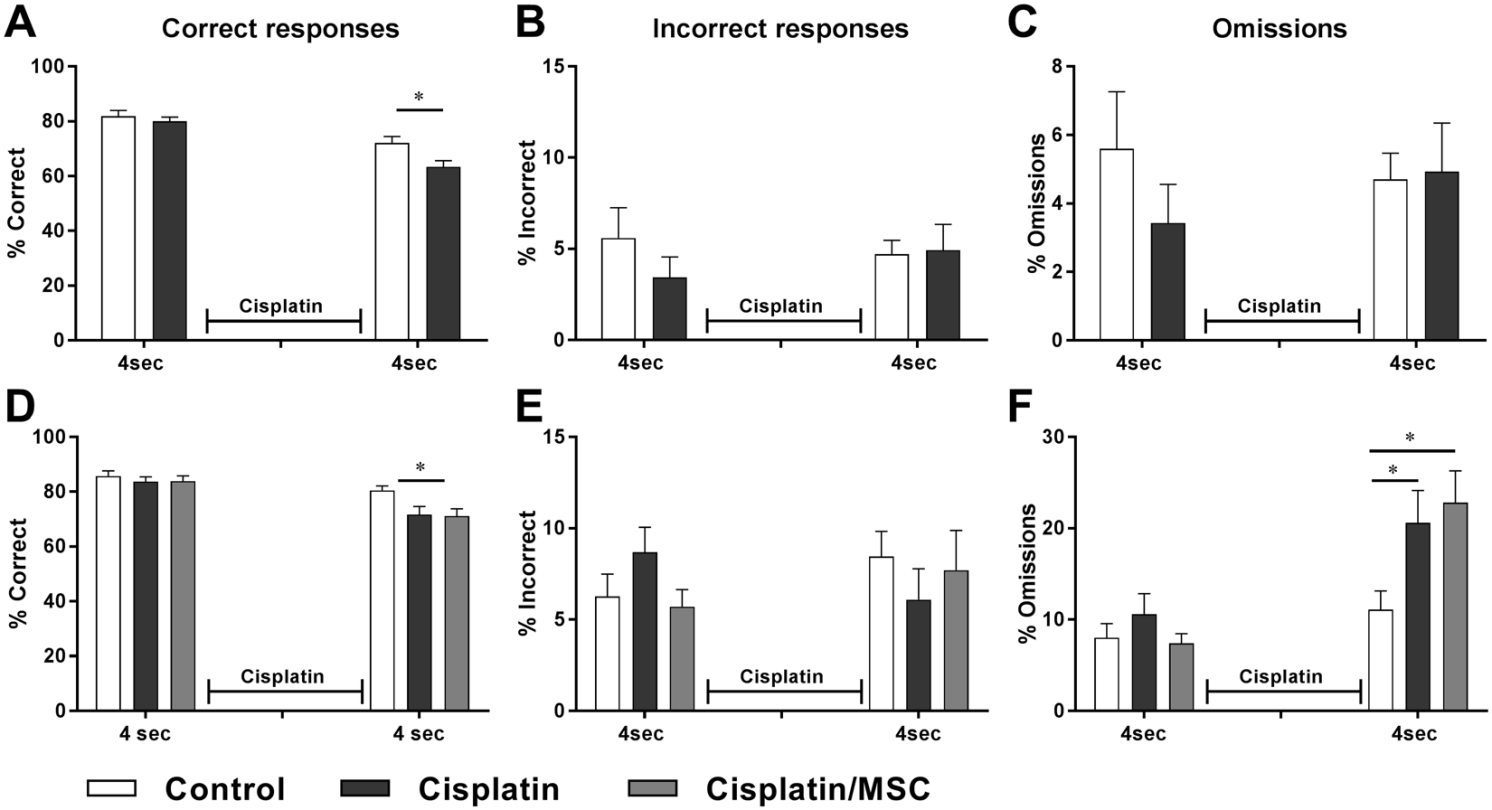
Cisplatin has an acute effect on performance in the 5CSRTT. Mice were trained to reach advancement criteria at a stimulus time of 4 seconds. Subsequently, mice were treated with cisplatin as in Fig. 1. Nine days after completion of cisplatin treatment, training in the 5CSRTT operant box resumed at a stimulus time of 4 seconds. Performance in the 5CSRT was assessed the first two sessions after the mice reached advancement criterion. The percentage of correct responses, omissions, and incorrect responses is presented. Data were analysed by 2-way ANOVA repeated measures followed by Tukey post-test. (**A**–**C**) Experiment 1 (control: n = 5; cisplatin: n = 7). (**D**–**F**) Experiment 2 (Control: n = 11; cisplatin: n = 10; cisplatin + MSC: n = 10). Correct responses: group × time interaction: *F* = 3.37; *P* < 0.05. Omissions: group × time interaction: *F* = 5.16; *P* < 0.05. *P < 0.05.

After completion of cisplatin treatment, we reintroduced the mice to the 5CSRTT at a stimulus time of 4 seconds according (Fig. 1B). On the second day, all mice had reached the advancement criteria (>50 responses, with >50% correct). However, the cisplatin-treated mice showed a lower percentage of correct responses (Fig. 2). The data in Fig. 2 indicate that this was due to an increase in omissions. There were no group differences in the percentages of incorrect responses. At this early time point after MSC administration, no improvement of performance in the 5CSRTT was observed (Fig. 2D–F).

### Effect of Cisplatin and MSCs on Performance in the 5CSRTT at a Short Stimulus Time

**Figure 3:**
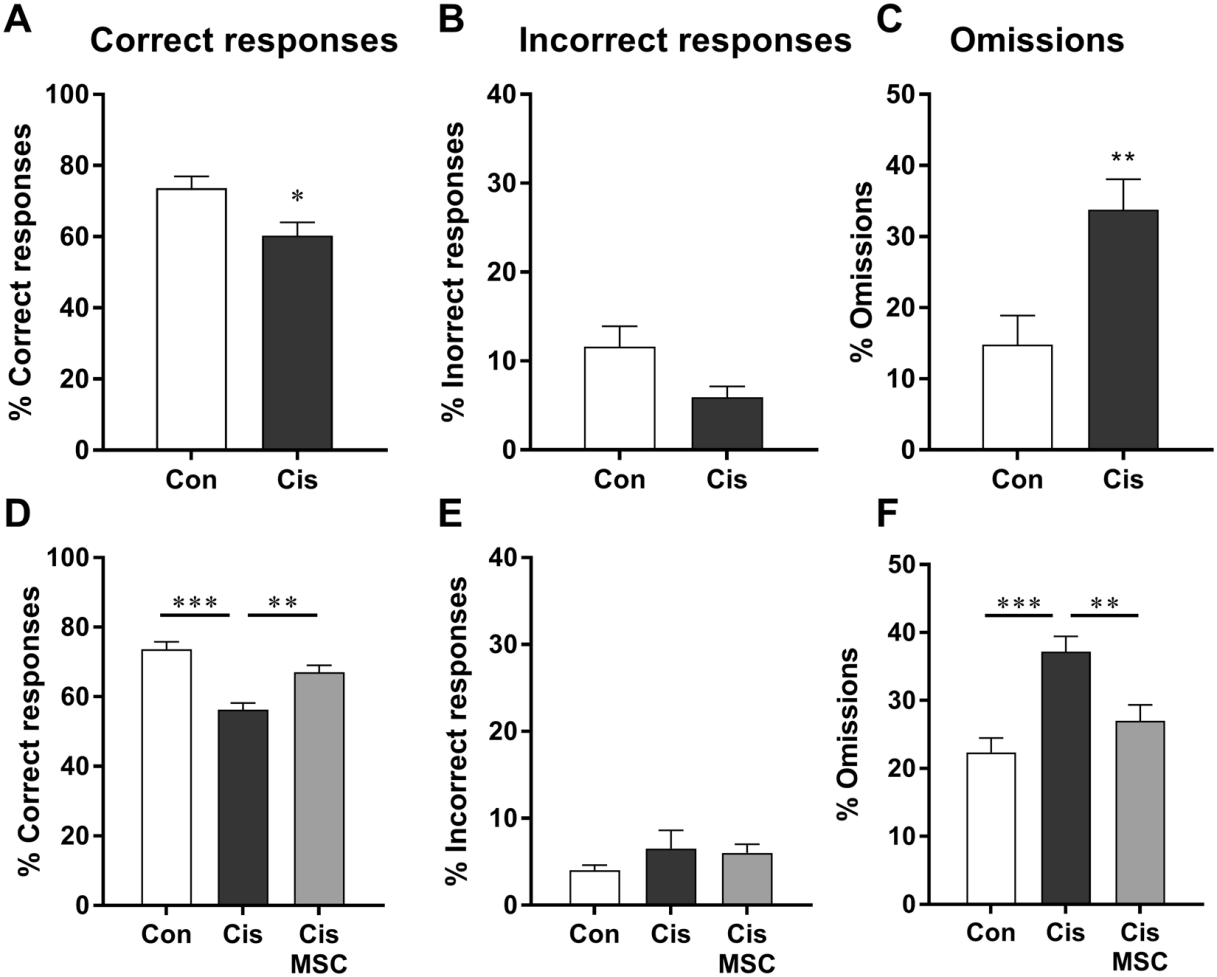
Cisplatin treatment induces a persistent increase in omissions that is reversed by MSC. Mice were treated with cisplatin and MSC as described in Fig. 1B and trained to advancement criteria at a stimulus time of 1 second. The percentage of correct responses, omissions, and incorrect responses was assessed. (**A**–**C**) Experiment 1 data were analysed with Student’s t-test. (**D**–**F**) Experiment 2 data were analysed with 1-way ANOVA followed by Tukey post-test. One-way ANOVA for correct responses: *F* = 18.56; *P* < 0.0001; 1-way ANOVA for omissions: *F* = 11.58; *P* < 0.001. *P < 0.05; **P < 0.01; ***P < 0.001.

Consistent with what we observed at the 4-second interval time, cisplatin-treated mice continued to show fewer correct responses at the 1-second interval time, and this was due to a larger percentage of omissions at both time points (Fig. 3A–F). These findings indicate that cisplatin treatment induces a persistent attention deficit. Notably, nasal administration of MSCs to cisplatin-treated mice normalized the number of correct responses and omissions (Fig. 3D–F).

**Figure 4:**
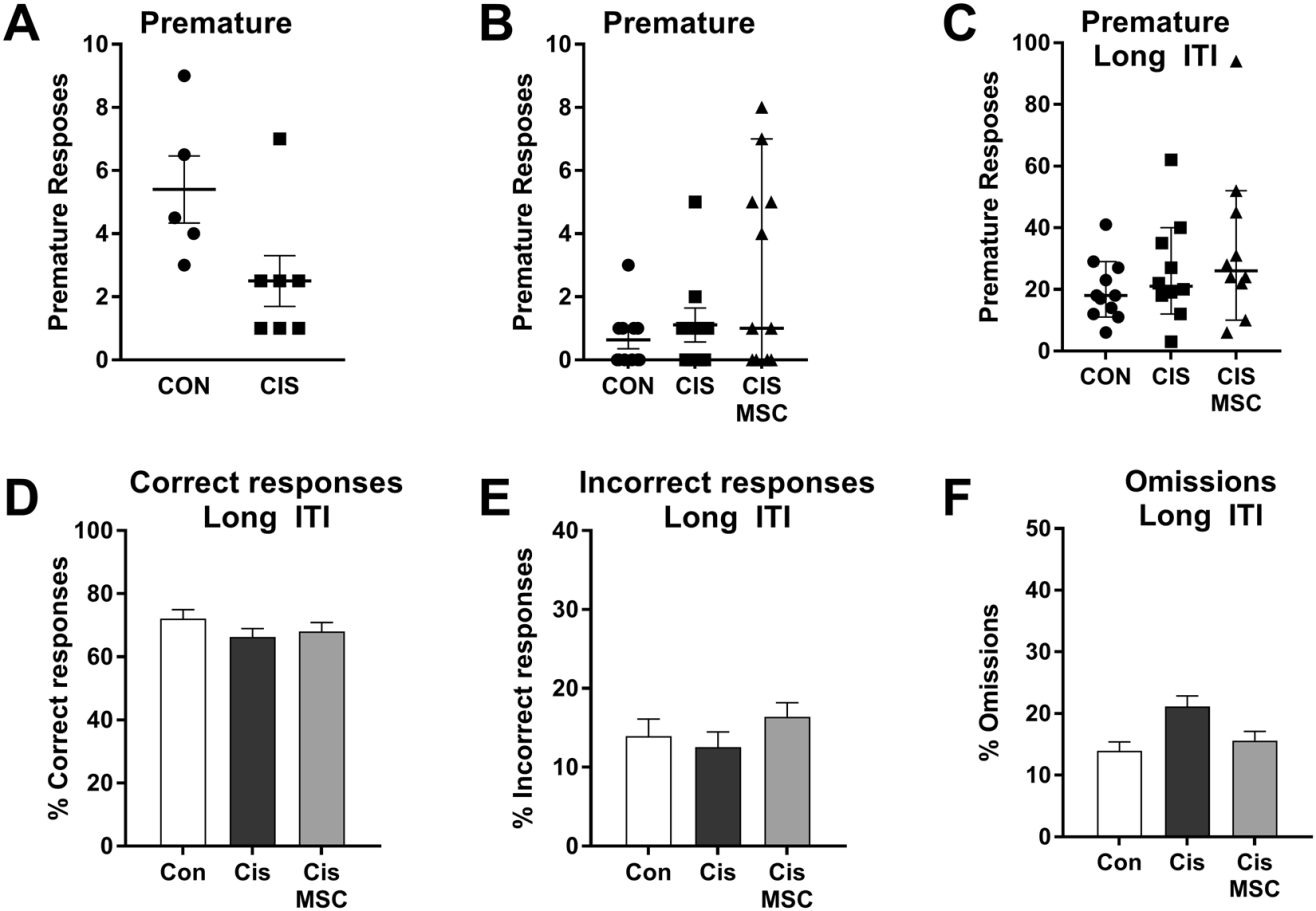
Cisplatin does not increase premature responses. Mice were treated with cisplatin and MSC as described in Fig. 1B and trained to advancement criteria at a stimulus time of 1 second. (**A**–**C**) The number of premature responses was quantified. (**A**) Experiment 1. (**B**) Experiment 2. (**C**) Experiment 2, ITI increased to 10 seconds. Data represent individual values and median +/−95% confidence. There were no statistically significant group differences detected. (**C**–**E**) Performance in the 5CSRTT at stimulus time of 1 second and ITI of 10 seconds was assessed for Experiment 2. No group differences in correct response, incorrect responses and omission were detected.

We did not detect any effect of cisplatin treatment on the number of premature responses in both experiments (Fig. 4A,B), indicating that the attention deficits (increased omissions) in cisplatin-treated mice were not associated with an increase in impulsivity. In the next set of trials in experiment 2, we increased the ITI from 5 to 10 seconds. As expected, this increased the number of premature responses (Figs 4C vs B). However, we still did not detect any effect of cisplatin on premature responses, supporting the notion that cisplatin treatment did not increase impulsivity (Fig. 4C). However, at the longer ITI we no longer detected group differences in correct responses and omissions (Fig. 4D–F), which further supports the conclusion that the increase in omissions is due to inattention^34^.

**Figure 5:**
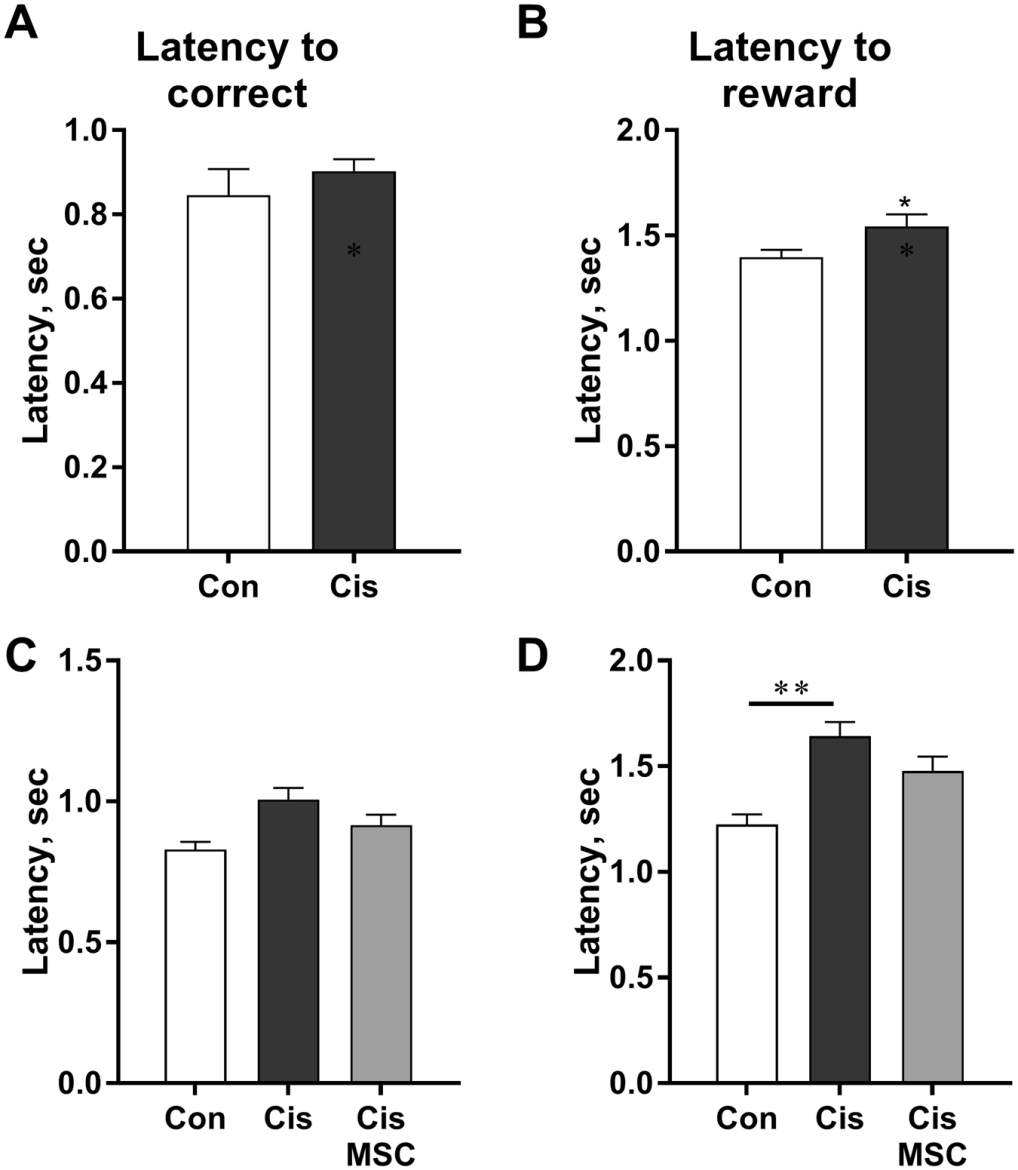
Effect of cisplatin and MSC on motivation. During the 5CSRTT described in Fig. 4, the latency to correct response (**A**,**B**) and to reward (**C**,**D**) was determined. (**A**,**C**) Experiment 1; **(B**,**D):** Experiment 2; Latency to reward: 1-way ANOVA *F* = 11.58; ***P < 0.001. *P < 0.05; **P < 0.01.

We also observed an increase in latency to reward in the cisplatin-treated mice (Fig. 5A,B) that was not affected by MSC treatment. An increase in the latency to reward may result from either lack of attention or a decrease in motivation. However, the latency to correct response, a measure frequently used to assess changes in motivation^35–38^, was not affected by cisplatin (Fig. 5C,D). Collectively, our findings indicate that cisplatin induced an increase in omissions that is mediated by a decrease in attention. However, we cannot fully exclude a contribution of decreased motivation.

**Figure 6:**
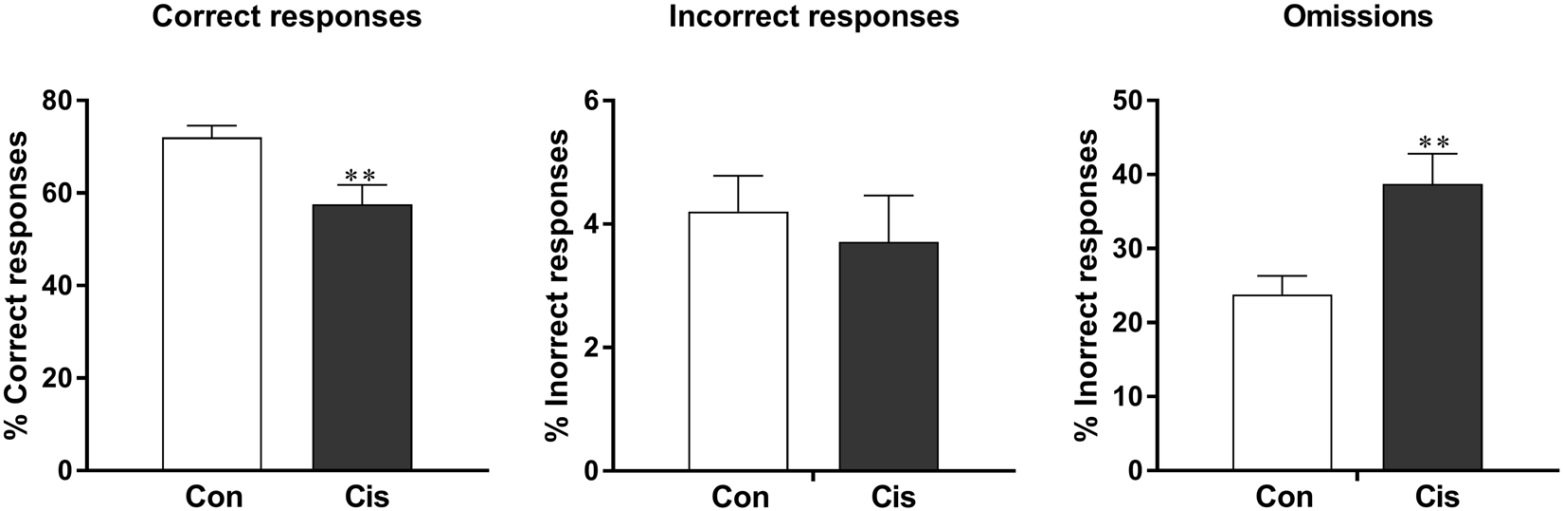
Cisplatin-induced deficit in attention is long-lasting. The mice of Experiment 1 were retrained and tested at 4 months after completion of cisplatin. The results represent data obtained at a stimulus time of 1 second. The percentage of correct responses, omissions, and incorrect responses was assessed and data were analysed by Student t-test. **P < 0.01.

To determine whether there is spontaneous recovery of the cisplatin-induced attention deficits, the mice in Experiment 1 were retested in the 5CSRTT starting 4 months after completion of treatment. Retesting the mice when they reached the advancement criterion at the 1-second stimulus time (approximately 8 weeks later) revealed that spontaneous recovery of cisplatin-induced attention deficits does not occur within this time frame (Fig. 6). The cisplatin-treated mice still displayed a decrease in correct responses that was fully explained by an increase in omissions.

### Effect of Cisplatin and MSCs on Synaptic Integrity

**Figure 7:**
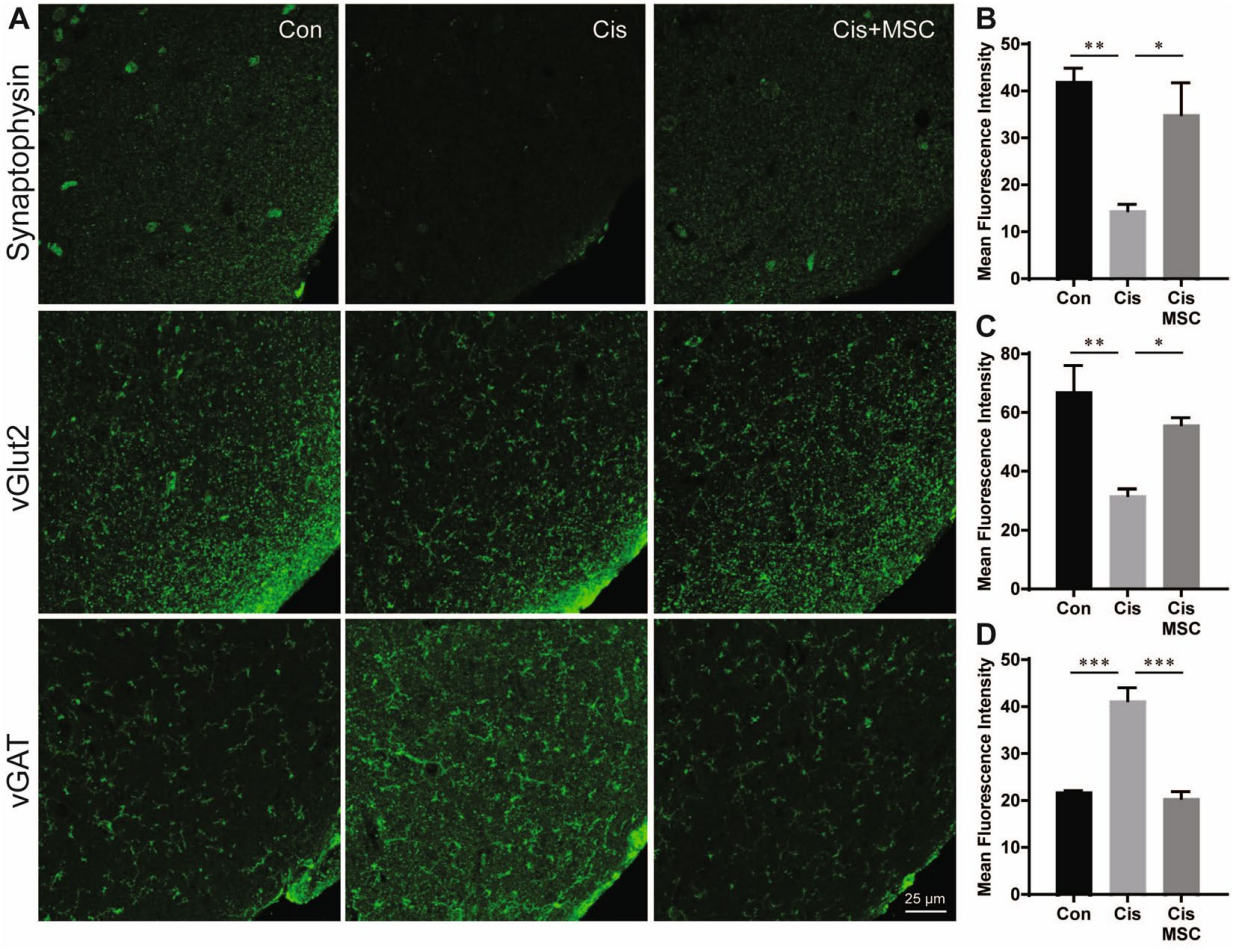
Cisplatin alters expression of presynaptic markers in the prefrontal cortex and this is reversed by MSC. The mice from Experiment 2 were sacrificed at an age of 11–12 months and expression of the presynaptic markers synaptophysin (**A**,**B**), vGlut2 (**C**,**D**), and vGAT (**E**,**F**) in the prefrontal cortex was assessed by immunofluorescence analysis. For details on the areas included in density analysis see Supplementary Fig. 1. Images are representative examples; bar graphs represent mean and SEM of 5 mice per group. ***P < 0.001; **P < 0.01; *P < 0.05.

To determine the effects of cisplatin and MSCs on synaptic integrity, we quantified the expression of the presynaptic markers synaptophysin, vGAT, and vGlut2, the postsynaptic glutamatergic marker PSD95, and the γ-aminobutyric acid (GABA)ergic marker gephyrin in the prefrontal cortex after completion of the behavioural assessments 4 months after the last dose of cisplatin, when the animals were 11–12 months old. Treatment of mice with cisplatin sharply reduced the level of synaptophysin (Fig. 7) in the prefrontal cortex. This reduction in synaptophysin was associated with a decrease in the presynaptic glutamatergic marker vGLUT2 and an increase in the presynaptic GABAergic marker vGAT (Fig. 7).

**Figure 8:**
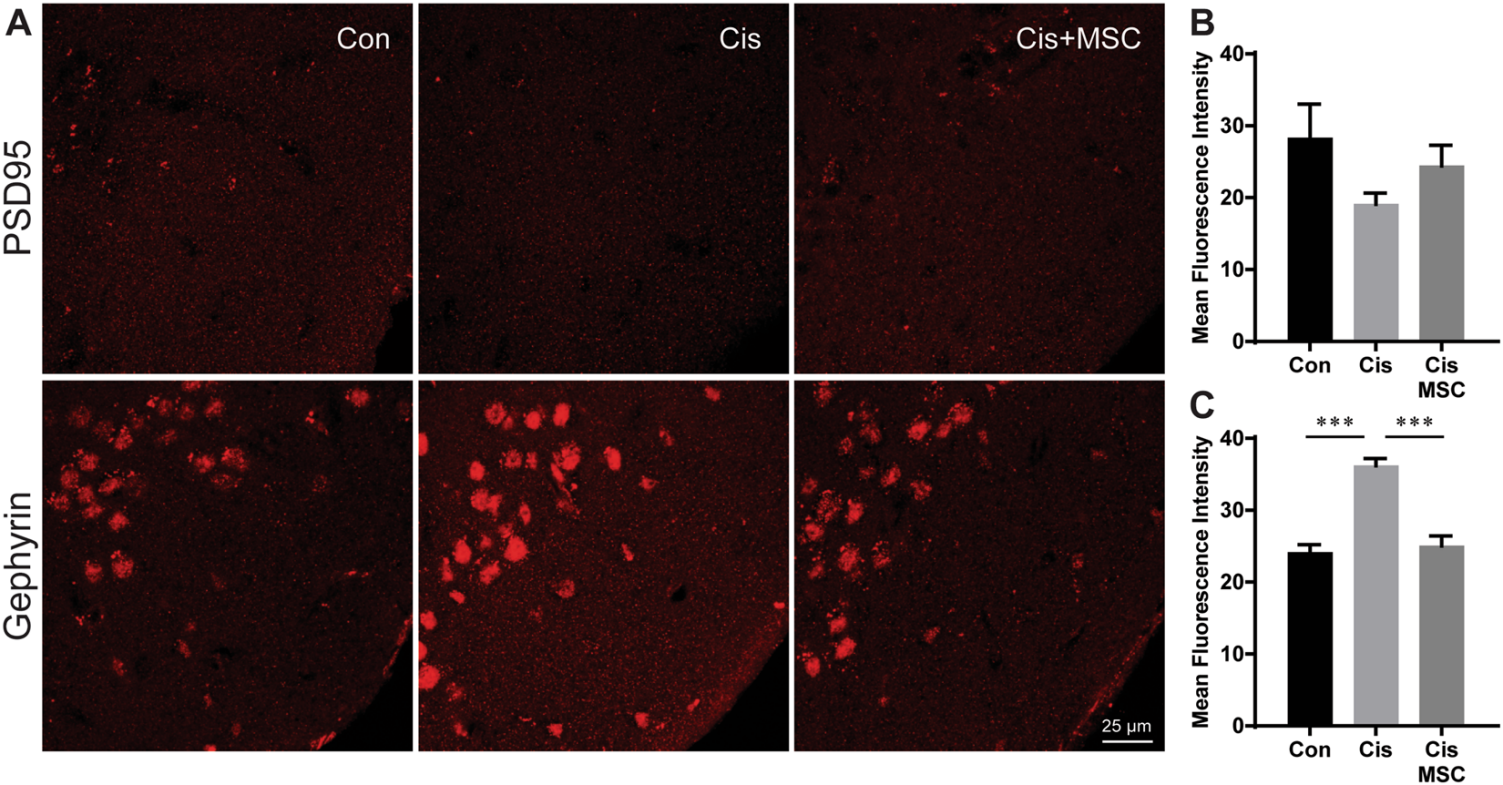
Effect of cisplatin and MSC on postsynaptic markers in the prefrontal cortex. The mice from Experiment 2 were sacrificed at an age of 11‒12 months. Expression of the glutamatergic postsynaptic marker PSD95 (**A**,**B**) and the GABAergic postsynaptic marker gephyrin (**C**,**D**) in the pre-frontal cortex was assessed by immunofluorescence analysis. We did not include the large bodies showing up in the gephyrin staining in our analysis. For details on the areas included in density analysis see Supplementary Fig. 1. Images are representative examples; bar graphs represent mean and SEM of 4‒5 mice per group. ***P < 0.001.

The change in the expression of the postsynaptic glutamatergic marker PSD95 did not reach statistical significance, while expression of gephyrin was increased in cisplatin-treated mice (Fig. 8). Nasal application of MSCs normalized the expression of all markers.

## Discussion

Attention deficits and problems concentrating are frequently reported by patients during and after cancer treatment^2–7^. Here, we examined the effects of cisplatin on the performance of adult male mice in the 5CSRTT to assess deficits in attention and response inhibition^24–28^. Our results clearly demonstrate that treatment of adult (7‒8 months old) male mice with cisplatin induces a deficit in attention, demonstrated by increased omissions in the 5CSRTT. Importantly, we also show that nasal administration of MSCs after completion of cisplatin treatment reversed the attention deficit. The attention deficits as detected in the 5CSRTT were associated with abnormalities in the expression of markers of synaptic integrity in the prefrontal cortex, as characterized by a reduction in expression of the presynaptic marker synaptophysin and the glutamatergic marker vGlut2 and an increase in expression of the presynaptic GABAerigc marker vGAT. MSC treatment normalized expression of these markers of synaptic integrity in the prefrontal cortex of cisplatin-treated mice. Further studies are needed to determine whether cisplatin and MSCs have similar effects in female mice.

We recently demonstrated that treatment of young adult male or female mice (8‒10 weeks old at start of treatment) with cisplatin reduced performance in a number of behavioural tasks that assess spatial orientation and short-term memory, including the social recognition task and the novel object and place recognition task, and the spontaneous alternations in the Y-maze test^21,22^ (and Chiu *et al*.^23^). In addition, we showed that cisplatin decreases the performance of young adult mice in the puzzle box, a task developed to assess executive functioning (Chiu *et al*.^23^). We also showed that nasal application of MSCs after completion of cisplatin treatment, when mice were 11‒13 weeks-old, normalized cognitive functioning in these tasks (Chiu *et al*.^23^). In the current study, we chose to use mice who fully had reached adulthood as this is more relevant from a translational viewpoint. We show here that cisplatin treatment of 7‒8-month-old mice induces deficits in attention evidenced by an increase in omissions in the 5CSRTT. Without further intervention, the attention deficit lasts until at least 4 months after completion of cisplatin treatment. Notably, we also show that nasal administration of MSCs after completion of cisplatin treatment (when the mice are approximately 8 months old) is sufficient to reverse the behavioural deficits. This is an important finding because it shows that MSCs are capable of promoting repair not only of the young adult brain, but also of the brains of mice that have fully reached adulthood.

Our data show that cisplatin treatment decreased the percentage of correct responses without changing accuracy, but rather by increasing omissions. This increase in omissions could be due to a decrease in motivation; however, we did not detect changes in latency to correct response, which would be expected if motivation were reduced. In addition, we do not have evidence for an increase in omissions toward the end of a session, when the mice are expected to become less motivated because they may become satiated (not shown). Cisplatin did not induce changes in premature or perseverant responses, indicating that the reduction in attention developed without evidence for hyperactivity.

The prefrontal cortex plays a key role in the regulation of attention^24,39^, and our data show that impaired attention in cisplatin-treated mice is associated with changes in markers of synaptic integrity in the prefrontal cortex. More specifically, we detected a reduction in the expression of the presynaptic marker synaptophysin and in the glutamatergic presynaptic marker vGlut2. In contrast, cisplatin increased expression of the presynaptic GABAergic marker vGAT. These findings indicate a change in the inhibitory/excitatory balance in the prefrontal cortex of cisplatin-treated mice. MSC treatment prevented this change in expression of markers of synaptic integrity. Studies in rats using specific GABA-A receptor agonists or antagonists have shown that both can lead to impaired attention characterized by increased omissions^40^, indicating that both increases as well as decreases in GABAergic activity could lead to the behavioural phenotype observed in our cisplatin-treated mice.

The schedule of cisplatin treatment used in our study induces signs of neuropathic pain^41–43^, and there is evidence that neuropathic pain is associated with changes in excitatory/inhibitory balance^44–48^. Moreover, creating an excitatory imbalance in the brain is sufficient to induce mechanical allodynia^49^. It is therefore possible that cisplatin-induced changes in the inhibitory/excitatory balance contribute to the signs of peripheral neuropathy.

It has been suggested that chemotherapy accelerates the aging process in the brain^50–52^. Synaptophysin expression decreases during aging, and although the postsynaptic protein PSD95 also is reduced during aging, it is retained longer at the synapse when age increases^53–55^. Moreover, in the prefrontal cortex, GABAergic activity increases with age while it lessens over time in other areas of the brain^56–59^. Thus, our finding that cisplatin induces a decrease in synaptophysin and an increase in vGAT without changes in PSD95 is consistent with the hypothesis that cisplatin treatment leads to accelerated aging of the brain. Further studies are needed to gain more insight into the effect of cisplatin and other chemotherapeutics on brain aging.

It remains to be determined via which mechanism cisplatin induces long-lasting changes in presynaptic and postsynaptic markers and how nasally administered MSCs exert their beneficial effects on performance in the 5-CSRTT and the associated deficits in expression of markers of synaptic integrity. We hypothesize that these long-lasting changes in synaptic integrity are the result of mitochondrial deficiencies, as has also been described for other neurodegenerative disorders^60,61^. This hypothesis is supported by our recent findings showing that cisplatin induces morphological abnormalities in synaptosomal mitochondria that are associated with functional mitochondrial deficits^21^ (and Chiu *et al*.^23^). We also presented evidence for a causal relationship between cisplatin-induced synaptosomal mitochondrial damage and cognitive impairment. Specifically, coadministration of the mitochondrial protectant pifithrin-μ prevented both mitochondrial abnormalities and cognitive deficits^21^. Moreover, the beneficial effect of MSC treatment on cognitive function in mice treated with cisplatin followed by MSCs at the age of 2‒3 months was associated with normalization of synaptosomal mitochondrial function (Chiu *et al*.^23^). RNAseq analysis of the effect of MSCs on gene expression in the hippocampi of mice treated with cisplatin revealed increased expression of genes involved in mitochondrial function and oxidative phosphorylation. These previous studies were performed in mice that were much younger than those in the current study (8‒10 weeks versus 7‒8 months) at the time of exposure to cisplatin. It remains to be determined whether the beneficial effects of MSCs that we detected in the present study are also mediated by restoration of mitochondrial function.

## Conclusions

Deficits in attention, focus, and concentration are frequently reported by patients with cancer who are treated with chemotherapy. In addition, objective tests of cognitive function in patients with chemobrain confirm the presence of attention deficits or concentration problems^2–7,13–18^. A recent imaging study in patients treated with cisplatin identified structural and functional alterations in the frontal and parietal regions of the cortex in patients treated for ovarian cancer with first-line taxane/platinum chemotherapy^62^. Here, we show that mice treated with cisplatin develop abnormalities in markers of synaptic integrity in the prefrontal cortex and deficits in attention, providing support for the clinical relevance of our model. Our findings that nasal application of MSCs after completion of cisplatin treatment normalizes both the deficits in attention as well as the abnormalities in synaptic integrity warrant further investigation and, potentially, clinical translation.

## Electronic supplementary material

Supplementary Figure 1

## Data Availability

The data that support the findings of this study are available from the corresponding author upon reasonable request.
