# Supplementary material for: Cisplatin treatment induces attention deficits and impairs synaptic integrity in the prefrontal cortex in mice
Source: Sci Rep. 2018 Nov 27;8:17400. doi: 10.1038/s41598-018-35919-x (PMC6258730; doi:10.1038/s41598-018-35919-x)
Supplement: Supplementary file 1 — Supplementary Figure 1 [file 41598_2018_35919_MOESM1_ESM.docx]

# SUPPLEMENTARY INFORMATION

## Cisplatin treatment induces attention deficits and impairs synaptic integrity in the prefrontal cortex in mice

Xiao-Jiao Huo, MSc^1^ xhuo@mdanderson.org

Teresa M. Reyes, PhD^2^ reyesta@ucmail.uc.edu

Cobi J. Heijnen, PhD^1,^* cjheijnen@mdanderson.org

Annemieke Kavelaars, PhD^1^ akavelaars@mdanderson.org

^1^ Neuroimmunology Laboratory, Department of Symptom Research, The University of Texas MD Anderson Cancer Center, Houston, TX, USA

^2^ Department of Psychiatry and Behavioral Neuroscience, College of Medicine, University of Cincinnati, Cincinnati, OH, USA

# SupplementaRY Figure 1


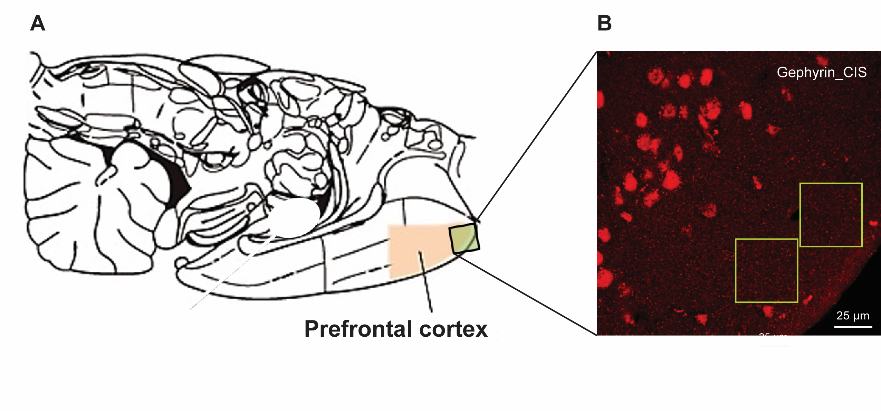


**A:** Schematic overview of sagittal section of the mouse brain indicating the area used for imaging in Figures 7 and 8. **B:** identification of the areas used for analyzing mean fluorescence intensity in all slides.
